# Design and Screening of M13 Phage Display cDNA Libraries

**DOI:** 10.3390/molecules16021667

**Published:** 2011-02-17

**Authors:** Yuliya Georgieva, Zoltán Konthur

**Affiliations:** 1 Department of Vertebrate Genomics, Max Planck Institute for Molecular Genetics, Ihnestraße 63-73, 14195 Berlin, Germany; 2 Department of Biology, Chemistry, and Pharmacy, Freie Universität Berlin, Takustraße 3, 14195 Berlin, Germany

**Keywords:** phage display, cDNA library, ORF selection, phagemid, protein-protein interaction, next generation sequencing (NGS)

## Abstract

The last decade has seen a steady increase in screening of cDNA expression product libraries displayed on the surface of filamentous bacteriophage. At the same time, the range of applications extended from the identification of novel allergens over disease markers to protein-protein interaction studies. However, the generation and selection of cDNA phage display libraries is subjected to intrinsic biological limitations due to their complex nature and heterogeneity, as well as technical difficulties regarding protein presentation on the phage surface. Here, we review the latest developments in this field, discuss a number of strategies and improvements anticipated to overcome these challenges making cDNA and open reading frame (ORF) libraries more readily accessible for phage display. Furthermore, future trends combining phage display with next generation sequencing (NGS) will be presented.

## 1. Introduction

Coupling of peptides to the surface of filamentous bacteriophages followed by affinity selection of binders to a chosen target was first described and termed “phage display” in 1985 by George P. Smith [1]. Since then, display of peptides and antibody fragments have been widely used, while the display of cDNA libraries remained problematic.

The most prominent and most frequently used system to date is the presentation of the desired molecules through coupling to the minor coat protein pIII of the filamentous phage M13. The pIII protein is positioned on one tip of the phage capsid and consists of three functional autonomous domains (D1, D2 and D3) joined by glycine rich linkers [2]. The D1-domain at the N-terminus is responsible for the translocation of the viral DNA into the host cytoplasm during infection of Gram-negative bacteria. The D2-domain binds to the bacterial F-Pilus and plays a central role in the infection process [3]. The C-terminal D3-domain is essential for the assembly of stable capsids [4] and a prerequisite for phage production. In most phage display vectors the pIII protein is missing the D1 and D2 domains resulting in phage-particles of reduced infectivity [5].

As the C-terminus of the shortened pIII has to remain intact to allow proper phage assembly, only N-terminal linkage of polypeptides to pIII is possible. Such constructs have been demonstrated to function well and display peptide epitopes [6,7], whole proteins [8], single chain antibodies [9] or Fab fragments [10]. However, expression of cDNA libraries in this fashion is challenging due to the naturally occurring translational stop codon in the 3’-region of reverse transcribed mRNAs. Moreover, the cDNAs, or fragments thereof, have to be in the same reading frame as the pIII protein, as well as the secretory leader sequence, and are not allowed to contain in-frame stop codons. One possibility to overcome this problem is to fragment the cDNA prior to its plasmid incorporation. But even though such inserts can lead to functional pIII production and a proper presentation of polypeptides, the majority of the clones contain non-functional inserts due to frame shifts or wrong orientation [11,12,13]. Hence, in most cases the poor display of cDNA expression products and the low percentage of target cDNAs being in the correct reading frames in the start library are hampering their application. Clearly, another limitation for efficient display of eukaryotic proteins on the phage surface is the poor ability of the *Escherichia coli* host system to perform post-translational modifications, which are in many cases essential for the proper folding and function of displayed proteins. However, the introduction of substrate phage display indicates possibilities to overcome these problems at least partially. Substrate phage display is designed to map acceptor sites for enzymatic reactions such as biotinylation, phosphorylation or phosphopantetheinylation, as reviewed by Yen and Yin [14]. The principle is to incubate a (poly)peptide phage display library with an enzyme of choice and to subsequently enrich the modified targets on a modification-specific selection matrix, such as an anti-phosphothyrosine antibody in case of a tyrosine kinase [15]. Recently, Aebi and co-workers demonstrated that asparagine-linked (*N*-linked) protein glycosylation can be achieved in *E. coli* by transfer of the glycosylation machinery of *Campylobacter jejuni* [16]. In a proof of principle experiment they showed that *in vivo* *N*-glycosylated proteins can be presented on phage particles. 

In the following sections, we will discuss a number of strategies and improvements employed to overcome the challenges associated with the generation of cDNA and open reading frame (ORF) phage display libraries. For instance, the use of cDNA fragmentation and ORF selection strategies prior initial library preparation leading to an increased expression of functional clones and improved presentation, as well as the application of different vector systems avoiding direct fusion to pIII. Furthermore, the use of comprehensive full-length ORFeome collections bypassing the application of conventional cDNA libraries and the implementation of next generation sequencing (NGS) technologies for fast and reliable identification of enriched binders will be discussed. While most of these considerations inevitably also apply to alternative phage display approaches, such as lambda and T7 display, we focus on the filamentous bacteriophage M13. Alternative phage display systems and their applications were discussed elsewhere [17,18,19].

## 2. Cloning Strategies for Functional cDNA Presentation

All five capsid proteins of the M13 bacteriophage have been successfully exploited for the presentation of fusion-proteins. For the display of proteins and peptides, N-terminal fusion to the minor coat protein pIII [1] and the major coat protein pVIII [20] proved most effective. Since pIII allows incorporation and presentation of larger inserts compared to pVIII, it is the scaffold of choice in most cases of ORF and protein display. Alternatively, C-terminal fusions to pVI for the display of cDNA libraries has been reported [21].

Besides the direct insertion of the gene of interest into the phage genome to generate a fusion to a coat protein encoding gene [9], phagemid vectors were established that combine selected genomic features of the phage with those of bacterial plasmids providing considerable advantages [22]. Phagemids contain both phage and bacterial origins of replication, a phage packaging signal, a selectable marker gene and the gene of the chosen coat protein for fusion. Its use allows easy preparation and maintenance of the vector, high yield of dsDNA and better transformation rates [23,24]. To assemble functional phage particles, co-infection with helper phage (e.g., M13K07) are required. The physical linkage between the phagemid encoded genotype and the displayed phenotype is achieved because helper phage have an origin of replication or a packaging signal of reduced functionality. Hence, phagemid vectors are being favorably incorporated into new viral particles rather than the helper phage genome and the resulting phage carry a mixture of wild-type and fusion coat proteins in a predominantly monovalent fashion [25]. For the purpose of multivalent display, alternative helper phage are required, such as the hyperphage. Hyperphage completely lack the gene for wild-type pIII in their genome and only pIII fusion-proteins are integrated into the viral particles [26]. This leads to reduced phage titers and reduced infectivity of the newly produced phage and therefore a combined application of both hyperphage and M13K07 during the biopanning process proved advantageous. Helper phage with a variety of different features have been discussed [27] and their efficiencies were compared [28].

### 2.1. Direct N-terminal fusion to pIII

The display of cDNA libraries as N-terminal fusion to pIII requires elimination of the naturally occurring stop codon and the removal of the 3’ untranslated region at the end of the cDNA. In addition, large cDNA fragments often show unsatisfactory presentation efficiencies compared to shorter inserts. To circumvent both problems, cDNA libraries are frequently fragmented prior to cloning assuming that in doing so functional binding domains are separated from potentially problematic sequences [12]. However, the majority of the clones in fragmented cDNA libraries appear to be non-functional or contain undesirable stop codons primarily as a result of the cDNA fragments being out of frame to the N-terminal leader sequence, or pIII, or both. To improve the quality of the fragmented cDNA library preparation, Zacchi and colleagues optimized the system by selecting for open reading frames prior cloning into phage display vectors [29]. Selection was achieved by cloning the fragmented cDNAs into a special vector flanking the potential ORF by a pIII-leader sequence at the N-terminus and the β-lactamase-gene at the C-terminus. Hence, only clones with in-frame inserts produced β-lactamase and could survive on ampicillin-containing agar plates. Since the β-lactamase gene was additionally flanked by loxP-sites, it was finally removed from the construct by Cre recombinase, resulting in a direct fusion of the ORFs with the pIII encoding gene [29,30]. Another group reported a similar strategy by which they simply subcloned the ORFs after positive selection on ampicillin instead of using the Cre-loxP recombination strategy [13].

An interesting way to bypass the ORF selection prior phage display library generation, but still obtaining ORF enriched cDNA libraries, was demonstrated by Hust and colleagues in 2006 [31]. The idea behind this strategy was deduced from the fact that intact phage particles can only be generated if pIII was available. Using hyperphage – which does not contribute wild-type pIII – instead of a conventional helper phage, all phagemid vectors containing stop codons in the cloned cDNA fragments instead of ORFs would result in the loss of pIII expression and, hence, would not generate phage particles. Indeed, 60% of all phagemids contained ORFs predicted to be immunogenic epitopes from *Salmonella typhimurium*, which was equivalent of a 10-fold enrichment.

### 2.2. Indirect fusion to pIII

**Figure 1:**
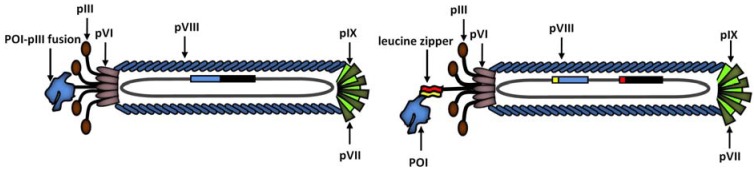
Schematic representation of bacteriophage M13 and different monovalent display types based on pIII coat protein. **(left)** Direct fusion of POI (protein of interest) to a truncated pIII. **(right)** Indirect fusion of POI to pIII by means of a leucine zipper structure.

A very elegant way to overcome the problems associated with the direct N-terminal fusion to pIII of cDNA libraries was developed and introduced by Crameri and Suter in 1993 [32]. They created a display system based on the strong natural bond of leucine zipper structures. Thus, the leucine zipper domains of the two transcription factors c-Jun and c-Fos were cloned in a single phagemid vector (pJuFo) in such a way that the Jun-leucine stretch was joined to the N-terminus of a truncated pIII and the Fos-leucine stretch was attached to the 5’-terminus of the cDNA fragments. Hence, the library contains primarily C-terminal fragments and full-length cDNA expression constructs. After their separate expression and transport to the periplasm, the two interaction partners meet and associate spontaneously forming a leucin zipper, thus providing the physical linkage between pIII and the protein to be displayed (Figure 1). Phage particles containing cDNA fragments in the wrong reading frame or possessing a premature stop codon will present short – mostly unnatural – peptides and/or phenotypically empty phage and will in most cases be lost during selection promptly.

The pJuFo system became quickly popular and is – to our knowledge – the most widely used system for displaying cDNA libraries on phage particles to date [19,33]. It has been applied for the selection and isolation of IgE-binders from various allergenic sources, such as *Aspergillus fumigatus* [34,35,36], peanut [37], dust mite [38], wheat [39] and many others [40,41]. Furthermore, this approach was successfully applied to recover known and novel autoantigens in autoimmune disorders, such as systemic lupus erythematosus [42] and vitiligo [43], as well as for example in prostate cancer [44]. 

A notably interesting application of the pJuFo system was reported 2002 by Brunet and colleagues [45]. The phage particles were decorated with the Stoffel fragment of DNA I polymerase (Taq) on the one hand and cross-linked with the substrate on the other. The library was then efficiently screened for catalytically active phage. They could demonstrate that indirect pIII-fusion can be applied for *in vitro* selections of enzymes with unknown catalytic activities from large protein libraries.

Recently, Weichel *et al.* investigated the presentation efficiency of the pJuFo system by expression of the *E. coli* alkaline phosphatase, PhoA [46]. Interestingly, the activity rate of the phage-displayed PhoA was identical to that of soluble PhoA, although it was shown that only dimeric PhoA is catalytically active [47]. This effectively demonstrated that also dimeric constructs can be properly presented using pJuFo phagemids without losing their functionality. As a consequence, further areas of application in the field of proteomics are conceivable, such as interactome analyses, investigation of protein complexes or exploration of enzyme-substrate relationships.

### 2.3. C-terminal fusion to pVI

The minor coat protein pVI is located at the same phage tip as pIII and is similarly available in three to five copies. It is the only coat protein with its C-terminus facing outwards and has its N-terminus buried in the phage coat [48,49]. Hence, direct C-terminal fusion of cDNA libraries for display is feasible and the presence of a stop codon in the cDNA structure do not hamper the expression of pVI and phage assembly. However, the same limitations in regard to presentation of out-of-frame peptides or empty phage apply as for the pJuFo system discussed above.

Its first use for screening C-terminally fused cDNA libraries was reported in 1995 by Jespers and colleagues [21]. The study describes the selection of novel serine protease inhibitors from a cDNA library of the pathogenic worm *Ancylostoma caninum*. Shortly after, the same research group generated a hybrid vector introducing a combination of lambda and pVI display [50]. To our knowledge however no other studies have implemented this system at present. 

In 1999, Hufton *et al.* reported on the construction of a set of vectors to fuse cDNA libraries to pVI in all three reading frames and their evaluation by selection of immunogenic ligands from a prostate cancer cDNA library [51]. Other examples include the selection of candidate tumor antigens using colorectal cancer sera [52] and the identification of collagen-binding proteins through screening of cDNAs from the human pathogen *Necator americanus* [53]. In summary, the application of pVI-display remains rare. Among other reasons, this is probably due to the generally lower display level of pVI-fused proteins in comparison to pJuFo presented proteins, as well as the reduced enzyme activity observed [46,51].

## 3. Different Pathways for Periplasmic Expression

An obvious obstacle for efficient presentation while working with highly heterogeneous libraries, such as cDNAs or ORFs, is the diverse nature of the molecules regarding their length, folding characteristics, stability or toxicity for the host. To limit these negative effects and minimize poor display, a number of strategies were assessed. For instance, the expression of wild-type pIII was reduced to increase the ratio of fusion proteins during phage assembly [54], the periplasmic folding of the target proteins was improved via chaperone co-expression [55,56]. Further, the amplification of non-displaying phage was reduced by utilizing an engineered helper phage lacking D1 and D2 domain of pIII resulting in loss of infectivity [57] or partially randomized signal sequences were employed to improve display efficiency [58]. Albeit helpful in many cases, these solutions still do not completely eliminate the encountered problems and a certain proportion of polypeptides remain refractory to display and will be eventually lost during the selection process. An additional measure to overcome presentation limitations was to employ different secretory pathways of the host in order to expand the range of properly presented and preferably functional polypeptides on phage.

**Figure 2:**
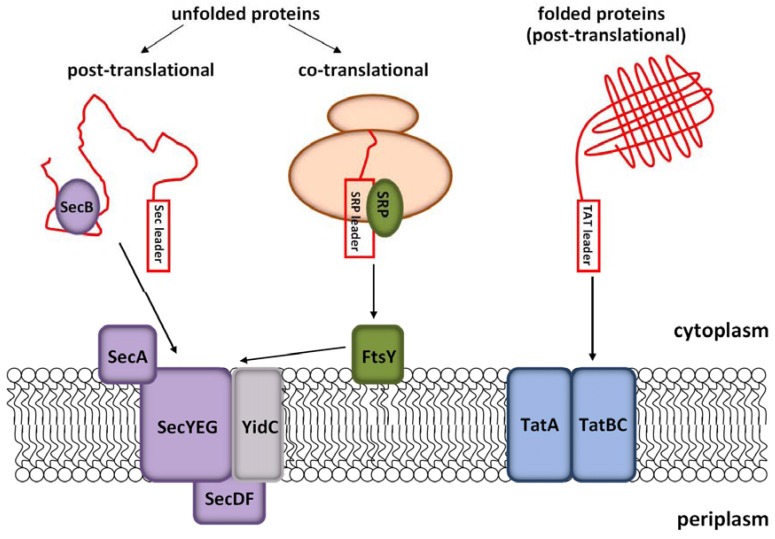
Schematic representation of the three major secretion pathways in *E. coli.*

In general, the display of proteins on the surface of filamentous bacteriophage relies on the efficient secretion of the protein of interest to the periplasm prior phage assembly. *E. coli* possesses three major pathways for the translocation of polypeptides across the cytoplasmic membrane to the periplasm: the signal recognition particle (SRP)-dependent Sec pathway for co-translational protein export, as well as the SRP-independent Sec pathway and the Tat (twin-arginine translocation) pathway for post-translational protein export (Figure 2). While proteins requiring periplasmic folding are targeted through the Sec and the SRP pathways, the Tat pathway is used for cytoplasmically folded polypeptides [59,60]. 

Targeting to the different translocation machineries is defined by the addition of specific leader peptides at the N-terminus of the protein of interest. An assessment of the literature on antibody phage display vectors has demonstrated that already a number of different leader peptides have been applied [61], most of them targeting the Sec pathway. One probable reason for this is that pIII can only fold correctly in the periplasm [62] and since antibody fragments (the most frequently displayed molecules) generally meet the requirements for the Sec translocon, other transport mechanisms were not systematically explored. Today, the most frequent Sec-dependent leader peptide in use is pelB, which was derived from the pectate lyase of *Erwinia caratovora*. For other molecules, such as DARPins, post-translational transport via the Sec pathway proved to be unsuitable, while co-translational transport via SRP lead to efficient display on the phage surface [63]. Recently, the display levels for single chain antibody fragment (scFv) using different Sec and SRP leader peptides were compared showing that the SRP pathway is equally applicable for antibody display [64]. 

In contrast to the Sec pathway, the Tat pathway can be employed for the translocation of folded proteins. Its use is essential for the display of polypeptides which need the reducing milieu of the cytoplasm and/or metal- or ATP-dependent cofactors and/or molecular chaperones for proper folding and biological activity. In 2005, Paschke and Höhne presented the Tat-mediated phage display system [65], which is based on the pJuFo. The protein to be displayed and pIII are expressed and translocated to the periplasm independently applying the Tat and Sec pathway, respectively, since pIII cannot be exported through the Tat-translocon. It’s application was effectively demonstrated by the display of functional green fluorescent protein of the jellyfish *Aequorea victoria* and mutants thereof on the phage surface, which require cytoplasmic folding to assemble the inner core chromophore. 

## 4. ORFeome Collections

The increasing availability of high-throughput technologies allowing to perform systematic analyses on whole-genome or proteome level has not only lead to a dramatic increase in experimental data but also in the number of resources becoming accessible. Since several years, the scientific community is pursuing the ambitious goal to assemble so-called ORFeome collections, which contain representative clones of full-length ORFs for all genes of an organisms. Currently ORFeome collections are being established for a wide range of organisms, among others for human [66,67], *Arabidopsis thaliana* [68], *E. coli* [69] as well as viral genes [70]. These collections are being gradually complemented by introducing new accessions, for instance splice variants, and some collections are available with and without stop codon at the end of the ORF sequence [66,67]. Most of the ORFeome collections are generated using the Gateway recombination technology, whereby cloning is performed without the need for restriction enzyme digest followed by ligation of the DNA to generate ENTRY clones for the respective ORFs [71]. Subsequently, they can be further transferred by recombination into any other vector containing complementary recombination sites. 

ORFeome collections represent a valuable source of well defined, homogeneous and ready to clone full-length cDNAs and we envisage that these can be made accessible to phage display applications soon. Combining selected subsets of clones with or without a stop codon from ORFeome collections with specifically modified phage display vectors of the types described above will allow the presentation of ORFs either as a direct or as an indirect fusion to the scaffold coat protein of choice. Undoubtedly, ORFeome phage libraries will be a powerful tool in future applications. Whether they will replace conventional cDNA libraries, however, needs to be seen. For the time being, cDNA libraries probably represent the anticipated *in vivo* situation of transcript diversity in a given organism or tissue thereof more precisely, since they are believed to cover a wider range of splice variants. 

## 5. Implementation of Next Generation Sequencing

The ever-increasing sequencing capacity of next generation sequencing (NGS) platforms, such as Roche/454-pyrosequencing or Illumina/Solexa Genome analyzer, revolutionized life sciences since its introduction and paved the way for completely novel approaches boosting biological and medical research [72]. 

Combining the power of NGS with the power of affinity driven selection strategies, such as phage display, has already started to change our way of thinking. Being able to sequence millions of DNA molecules at a time in practically only a few days has a major impact on the way selections and their evaluation can be performed. For instance, the diversity of initial libraries can be analyzed, as demonstrated by Pons and co-workers [73]. They have obtained more than one million sequences for their naïve scFv phage display library using Roche/454-pyrosequencing, which has allowed the analysis of immunoglobulin V-gene usage in their library. Furthermore, NGS made it possible to follow the enrichment process of displayed molecules during all rounds of selection. Dias-Neto *et al.* recently used the Roche/454-pyrosequencing platform to analyze the outcome of *in vivo* peptide library selections in cancer patients [74]. They additionally evaluated the outcome of the selection by sequencing large numbers of clones by the Sanger method and a comparison of these results with NGS revealed that ~80–95% of all sequences generated by traditional sequencing have also been recovered by NGS. In another recent report, Ravn *et al.* monitored the enrichment process over multiple rounds of selection with an antibody library in scFv format using the Illumina/Solexa Genome analyzer platform [75]. They could demonstrate that all positive clones identified by classical ELISA conducted at the end of a selection were also recovered by NGS. Yet some highly enriched clones were missed by conventional ELISA-screening but successfully identified by NGS. These clones were subsequently analyzed and found to be badly expressed in *E. coli* as soluble antibody fragments and hence escaped the ELISA screening. 

Applying a phage display library of fragmented human cDNA, Di Niro *et al.* conducted a protein-protein interaction screen for the identification of interaction partners of human transglutaminase 2 (TG2) [30]. Next to the isolation of known and novel interaction partners, the use of NGS for the evaluation of the enriched binder pool led to the identification of the specific domain of one of the candidates directly involved in the interaction with TG2. 

Beyond doubt, next generation sequencing technologies will be increasingly employed to many different phage display applications in future, as NGS provides invaluable information about all stages of the selection procedure in a fraction of a time compared to standard evaluation methods currently applied. 

## 6. Conclusions and Outlook

In the last decades, numerous combinatorial methodologies for the selection and screening of proteins were developed, which mimic natural evolutionary processes *in vitro* and aim at detecting interaction partners with specific chemical and/or physical properties. Among these, surface display technologies play a central role and besides bacterial, ribosomal, yeast and other screening methods, phage display is by far the most widespread application for screening of protein interaction partners or selecting antibodies for diagnostic and therapeutic applications [76]. 

With the latest developments discussed here, the presentation of cDNA and ORF expression products on phage has become not only possible, but led to an increase in library quality, produced in-depth analysis of the enrichment process, became faster, more convenient and reasonably priced. Thus the application of this approach and variations thereof will increase constantly in the future. Most application areas described in this review centre around the identification of novel allergens, potential biomarkers in autoimmune disorders [43,77], cancer [78,79] or vaccine development [80]. However, additional fields of application, such as screening for cellular targets of toxic chemical compounds emerge [81].

The next level of phage display will indisputably see the implementation of NGS into automation strategies for screening of combinatorial libraries [82]. The application of semi-automated selection strategies, such as developed in our laboratory [83,84] not only allows the reduction of variability and increased reproducibility of the selection protocol, but can be readily implemented in process pipelines based on unit-automation [85,86]. Such pipelines, in turn, can be easily extended to include NGS as a sophisticated analysis tool of the selection outcome. 

Twenty-five years after its introduction, phage display persists to be the most popular and successful surface display application with more than 4,000 entries in PubMed. With the latest developments in library design and the combination of phage display with high-throughput selection pipelines and next generation sequencing, the field has become as vibrant as ever. 
